# 

**Published:** 1866-04

**Authors:** 


### CONTENTS.

### ORIGINAL CONTRIBUTIONS.

ART. XIII. THE PATHOLOGY AND TREATMENT OF EPIDEMIC CHOLERA. By N. S. DAVIS, M.D., Prof. Practical and Clinical Medicine, Chicago Medical College, ---- 193

ART. XIV. BLOOD-LETTING IN SYMPTOMATIC FEVERS By S. R. MILLARD, M.D., Aurora, Ill., ---- 198

ART. XV. SEVERE AFFECTION OF AN EYE, RESULTING FROM A DISEASED TOOTH, Reported by JOS. S. HILDRETH, M.D., late Surgeon in charge of the U.S. Army Eye and Ear Hospital, Chicago, ---- 201

### PROCEEDINGS OF SOCIETIES.

Proceedings of Quincy Medical Society, ---- 203

Proceedings of a Meeting of the County Medical Societies of Northern Indiana, ---- 210

### SELECTIONS.

On the Pathology and Treatment of Spermatorhœa. By Roberts Bartholow, M.D., ---- 212

The Use of the Artificial Membrana Tympani. By D. B. St. John Roosa, M.D., Aural Surgeon to the New York Eye and Ear Infirmary, &c., 223

The Rinderpest, ---- 227

### FOREIGN CORRESPONDENCE.

Letter from John M. Woodworth, ---- 230

### BOOK NOTICES.

A Treatise on the Principles and Practice of Medicine; designed for the Use of Practitioners and Students of Medicine. By Austin Flint, M.D., 233

The Malformations, Diseases, and Injuries of the Fingers and Toes, and their Surgical Treatment. By Thomas Annandale, F.R.C.S.E., ---- 234

Lectures on Epilepsy, Pain, Paralysis, and Certain other Disorders of the Nervous System. By Charles Bland Radcliffe, M.D., ---- 234

The Physiology of Man. By Austin Flint, Jr., M.D., ---- 235

A Practical Treatise on Urinary and Renal Diseases, including Urinary Deposits. By William Roberts, M.D., ---- 235

Extract from an Unpublished Essay on Physical Force. By L. Mackall, 235

An Essay on the Life in Nature. By Louis Mackall, M.D., ---- 235

An Essay on the Law of Muscular Action. By Louis Mackall, M.D., ---- 235

### EDITORIAL.

American Medical Association, ---- 235

Lecture Fees in Medical Colleges, ---- 236

Chicago Medical Society, ---- 240

Mercy Hospital, ---- 245

Deaths, ---- 246

Diseases of the Eye and Ear in the County Hospital, ---- 246

Graduates, ---- 246

Disease of the Eye, connected with a Diseased Tooth, ---- 247

London Lancet, ---- 247

American Medical Association, ---- 247

Mortality for the Month of February, ---- 248

Annual Report of the Insane Hospital of California, ---- 250

Treatment of Asiatic Cholera, ---- 251

The Therapeutic and Physiological Action of Digitalis, ---- 252

Discovered Remedy for certain forms of Neuralgia, ---- 253

Dr. James P. White, of Buffalo, ---- 253

M. Jobert de Lamballe, ---- 253

Dr. T. G. Thomas, of New York, ---- 253

